# A shallow morphology of the intertubercular groove is associated with medial and bilateral but not lateral pulley lesions

**DOI:** 10.1007/s00167-023-07350-x

**Published:** 2023-02-23

**Authors:** Benjamin Daniel Kleim, Jose Fernando Sanchez Carbonel, Maximilian Hinz, Marco-Christopher Rupp, Bastian Scheiderer, Andreas Balthasar Imhoff, Sebastian Siebenlist

**Affiliations:** Department of Sports Orthopedics, Technical University of Munich, Ismaninger Str. 22, 81675 Munich, Germany

**Keywords:** Biceps reflection pulley, Rotator cuff, Intertubercular groove morphology, Dysplastic, Influencing factor

## Abstract

**Purpose:**

To investigate the influence of intertubercular groove (IG) morphology on the development of different types of biceps reflection pulley (BRP) injuries.

**Methods:**

A consecutive cohort of 221 patients with ventral shoulder pain and a preoperative diagnosis suspecting BRP injury, who underwent arthroscopy, was retrospectively reviewed. The presence or absence as well as type of pulley injury (medial, lateral or bilateral) was confirmed arthroscopically. The intertubercular groove was evaluated on MRIs after triplanar reconstruction of the axial plane. IG depth, width, medial wall angle (MWA), lateral wall angle (LWA) and total opening angle (TOA) were measured. IG depth and width were expressed in relation to the humeral head diameter. Measurements were performed by two clinicians independently and averaged.

**Results:**

Of 166 included patients 43 had bilateral, 65 medial and 38 lateral BRP lesions. 20 patients had intact BRPs and represented the control group.

The intra-class correlation coefficient of measurements was 0.843–0.955. Patients with a medial or bilateral BRP injury had a flatter MWA (38.8° or 40.0° vs. 47.9°, *p* < 0.001), wider TOA (96.1° or 96.6° vs. 82.6°, *p* < 0.001), greater width (12.5 or 12.3 vs. 10.8 mm, *p* = 0.013) and shallower depth (5.5 or 5.4 vs. 6.2 mm, *p* < 0.001) than the control group. Conversely, the IG morphology of those with lateral BRP injuries did not differ significantly from the control group. The odds ratio for a medial or bilateral BRP injury when the TOA exceeded 95° was 6.8 (95% confidence interval 3.04–15.2).

**Conclusion:**

A dysplastic type of IG morphology with a wide TOA, flat MWA, decreased depth and increased width is associated with the presence of medial and bilateral BRP injuries. A TOA of > 95° increases the likelihood of a medial or bilateral BRP injury 6.8-fold. Lateral BRP injuries are not associated with dysplastic IG morphology. Concomitant LHBT surgery may, therefore, not always be necessary during isolated supraspinatus tendon repair.

**Level of evidence:**

Level III.

## Introduction

The biceps reflection pulley (BRP) comprises the superior glenohumeral ligament (medially) and the coracohumeral ligament (laterally), as well as the adjacent rotator cuff (RC) tendons subscapularis (SSC) and supraspinatus (SSP) [12, 15, 18, 22, 23]. This soft tissue structure helps to stabilise the long head of biceps tendon (LHBT) before it enters the bony intertubercular groove (IG) [12, 15, 18, 22, 23].

Lesions of the biceps reflection pulley (BRP) are present in 45–90% of shoulders with rotator cuff tears [13]. These can be classified into medial, lateral and bilateral lesions [23]. BRP lesions are responsible for morbidity associated with RC tears as well as pathology of the LHBT [4, 13, 18]. BRP lesions are associated with instability of the LHBT in the IG [18, 31, 32]. This instability may be a result of the loss of the soft tissue stabilizers, or also be a cause of the tear of the soft tissue structures of the BRP [12, 22, 31]. For this reason, LHBT tenotomy or tenodesis is often performed concomitantly during RC repair, although it is still unclear for which patients this is necessary [8, 11, 24, 30, 35].

Increased medial shear force vectors acting on the LHBT have been described in various physiological positions, however the precise mechanism of injury of the BRP is unclear [7]. Medial displacement of the LHBT in the IG has been suggested as a sign of a BRP lesion on MRI, however preoperative diagnosis is challenging and arthroscopy remains as the gold standard [26].

The bony IG provides a channel for the LHBT on the proximal humerus and the morphology of this has been found to vary and influence the development of tendinitis and medial dislocation of the LHBT [10, 16, 34]. However, the influence of the morphology of the IG on the development and type of BRP lesions is yet unknown.

The purpose of this work was to investigate the influence of intertubercular groove (IG) morphology on the presence of different types of biceps reflection pulley (BRP) injuries.

The hypothesis was that IG depth and wall angles would differ between 4 subgroups (patients with intact BRPs or with medial, lateral, or bilateral BRP injuries), being shallower in the direction of the injury.

## Materials and methods

**Fig. 1:**
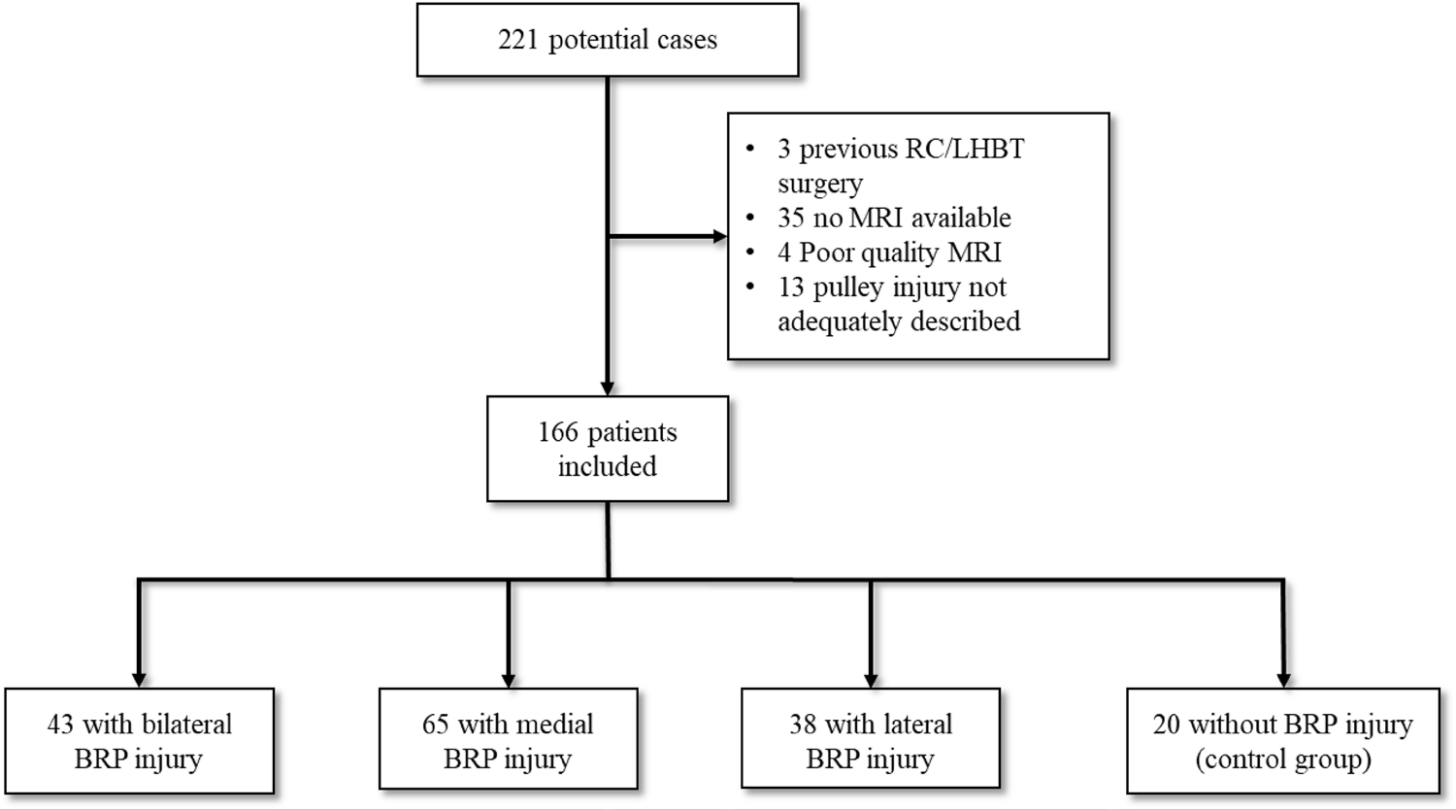
After applying the exclusion criteria patients were assigned to one of 4 subgroups based on the findings at arthroscopy

Institutional review board approval was granted by the ethics committee of the medical faculty of the technical university of Munich, Germany (reference 359/20 S). All 221 patients, who were preoperatively suspected of having a BRP injury and subsequently underwent arthroscopic shoulder surgery at our department between February 2012 and December 2018 were retrospectively considered for inclusion. The preoperative diagnosis of BRP injury was based on a history of anterior shoulder pain and positive clinical examination for LHBT pathology (palpatory pain at the IG, positive Speed’s test) with suggestive findings on MRI (medial displacement or even dislocation of the LHBT and/or a tear of the cranial SSC tendon or the anterior SSP Tendon and/or effusion around the LHBT) [5, 26]. Inclusion criteria were a suspected BRP injury as the preoperative diagnosis (with or without a tear of the cranial SSC tendon or anterior SSP tendon) and the availability of preoperative MRI images of adequate quality for reliable measurement, taken no more than 4 months prior to surgery. Exclusion criteria were previous surgery to the LHBT or BRP. The existence and type of BRP lesion was documented in detail in the operative report. Where this was not clearly described these patients were excluded from the investigation (Fig. 1).

### Arthroscopy findings

**Fig. 2:**
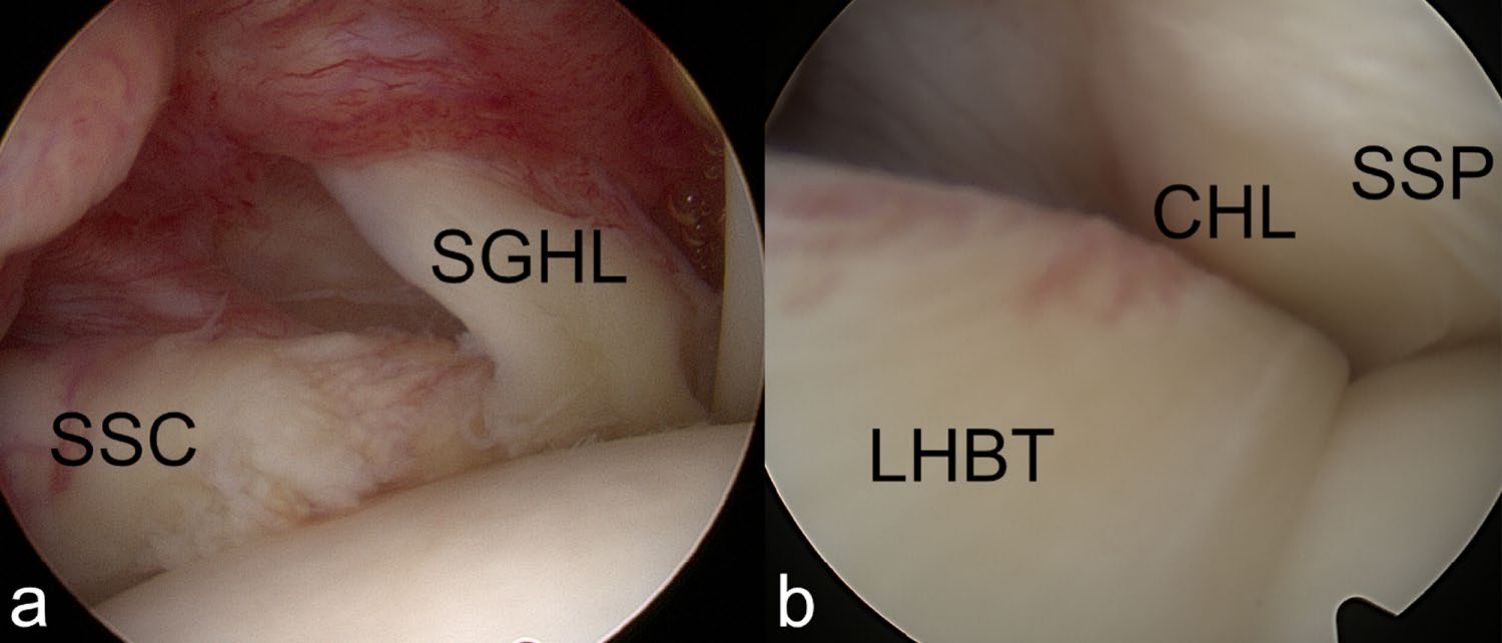
Arthroscopic images of a medial BRP injury around the long head of biceps tendon (LHBT). The medial part (**a**) shows a tear of the superior glenohumeral ligament (SGHL) and a tear of the cranial subscapularis tendon (SSC). The lateral BRP (**b**), including the coracohumeral ligament (CHL) and supraspinatus tendon (SSP), is intact

**Fig. 3:**
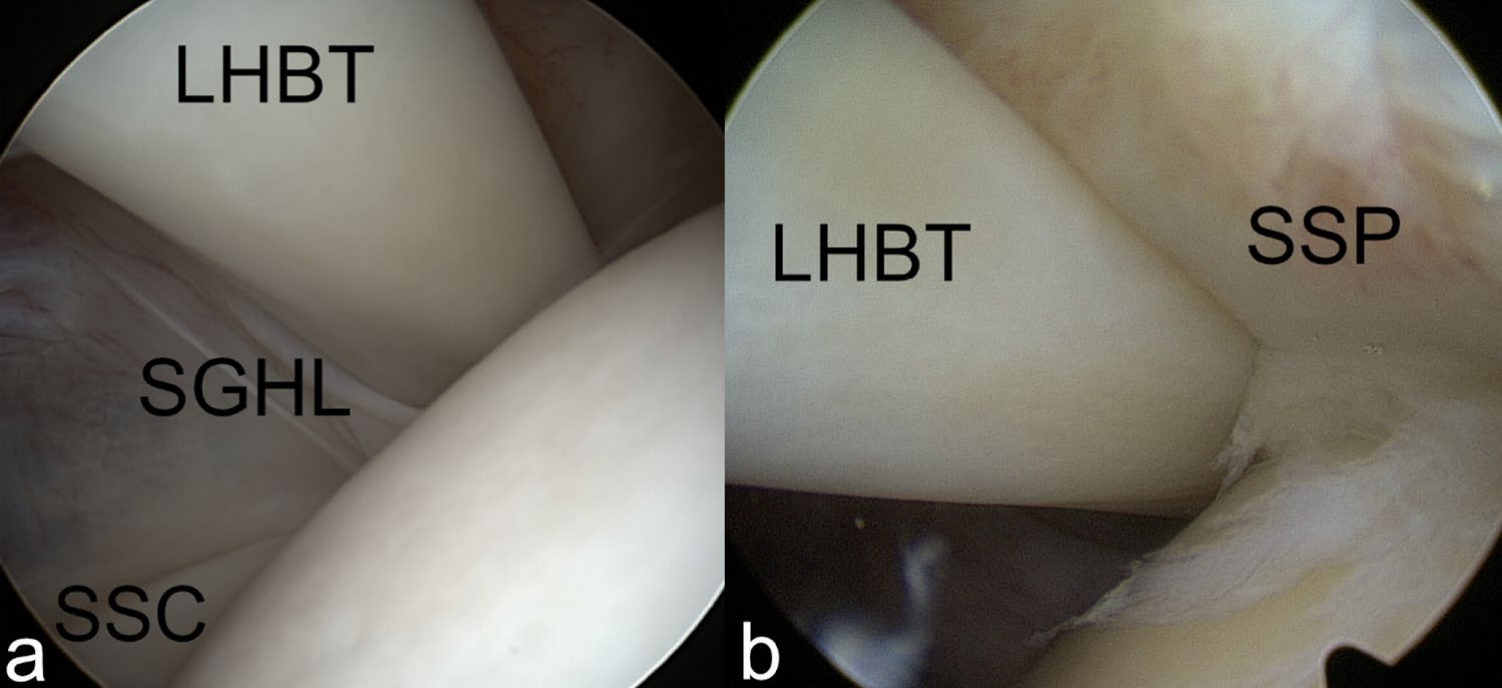
Arthroscopic images of a lateral BRP injury around the long head of biceps tendon (LHBT). The medial part (**a**), comprising the subscapularis tendon (SSC) and superior glenohumeral ligament (SGHL), is intact. Laterally (**b**) the coracohumeral ligament is torn and a partial articular sided avulsion of the supraspinatus tendon (SSP) is visible

**Fig. 4:**
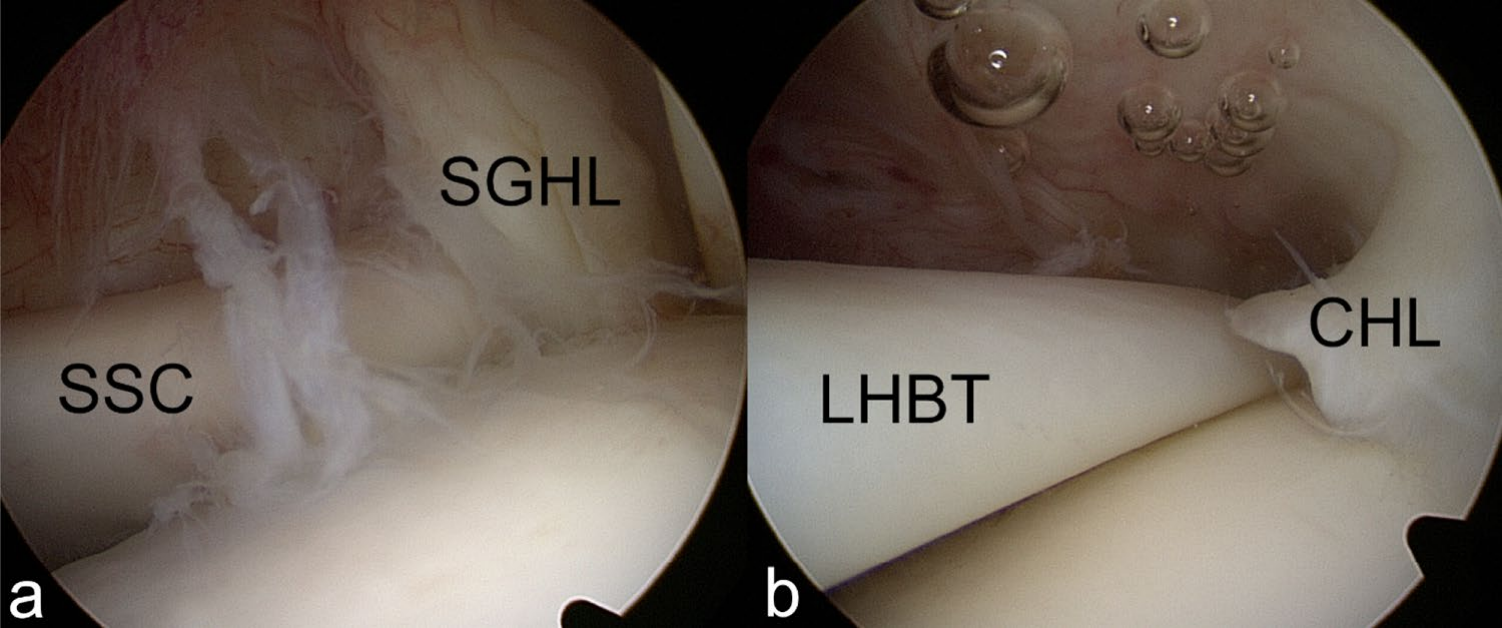
Arthroscopic images of a bilateral BRP injury around the long head of biceps tendon (LHBT). The medial part (**a**) shows a tear of the superior glenohumeral ligament (SGHL) and partial avulsion of the cranial subscapularis tendon (SSC). The lateral part (**b**) shows a tear of the coracohumeral ligament (CHL)

**Fig. 5:**
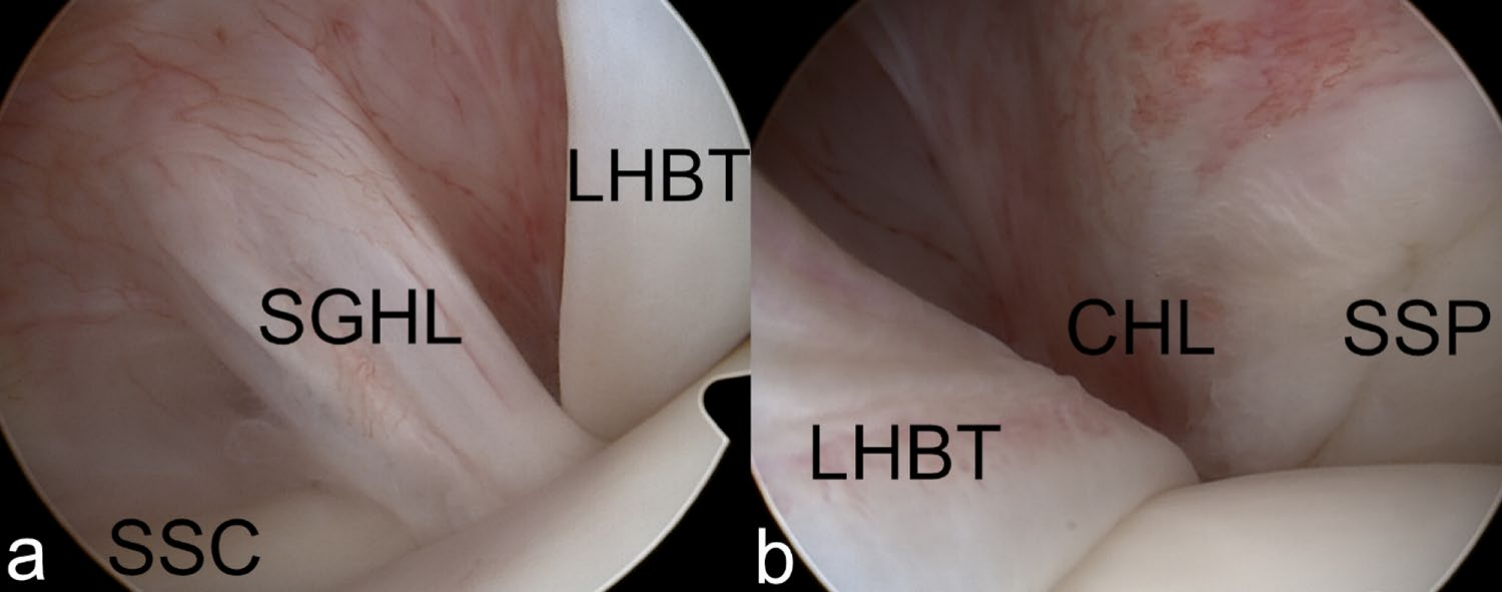
Arthroscopic images of an intact BRP around the long head of biceps tendon (LHBT). Medially (**a**) the superior glenohumeral ligament (SGHL) and subscapularis tendon (SSC) are intact. Laterally (**b**) the coracohumeral ligament (CHL) and supraspinatus tendon (SSP) are intact

Where an injury to the BRP was confirmed intraoperatively, lesions were classified as medial (Fig. 2), lateral (Fig. 3) or bilateral (Fig. 4) injuries according to the findings from the operative report [23]. Shoulders which were found to have an intact BRP (Fig. 5) were assigned to the control group.

### Morphology of the intertubercular groove

**Fig. 6:**
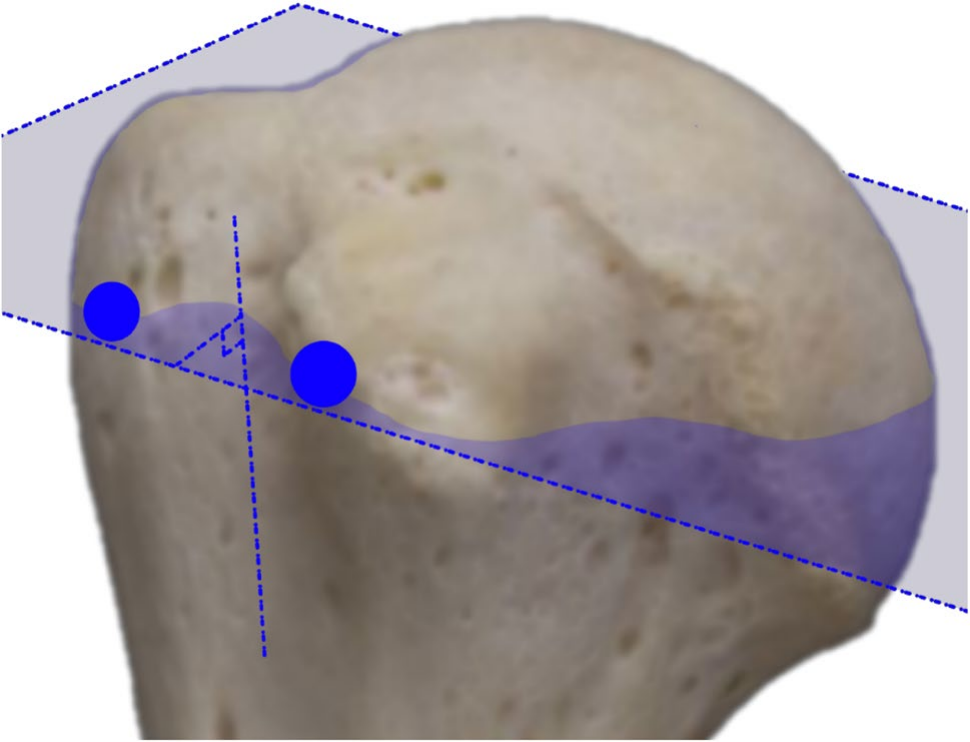
The axial plane was reconstructed in 3 planes, to be at the level of and in line with the highest point of each tuberosity, whilst being aligned at 90° to the floor of the IG

**Fig. 7:**
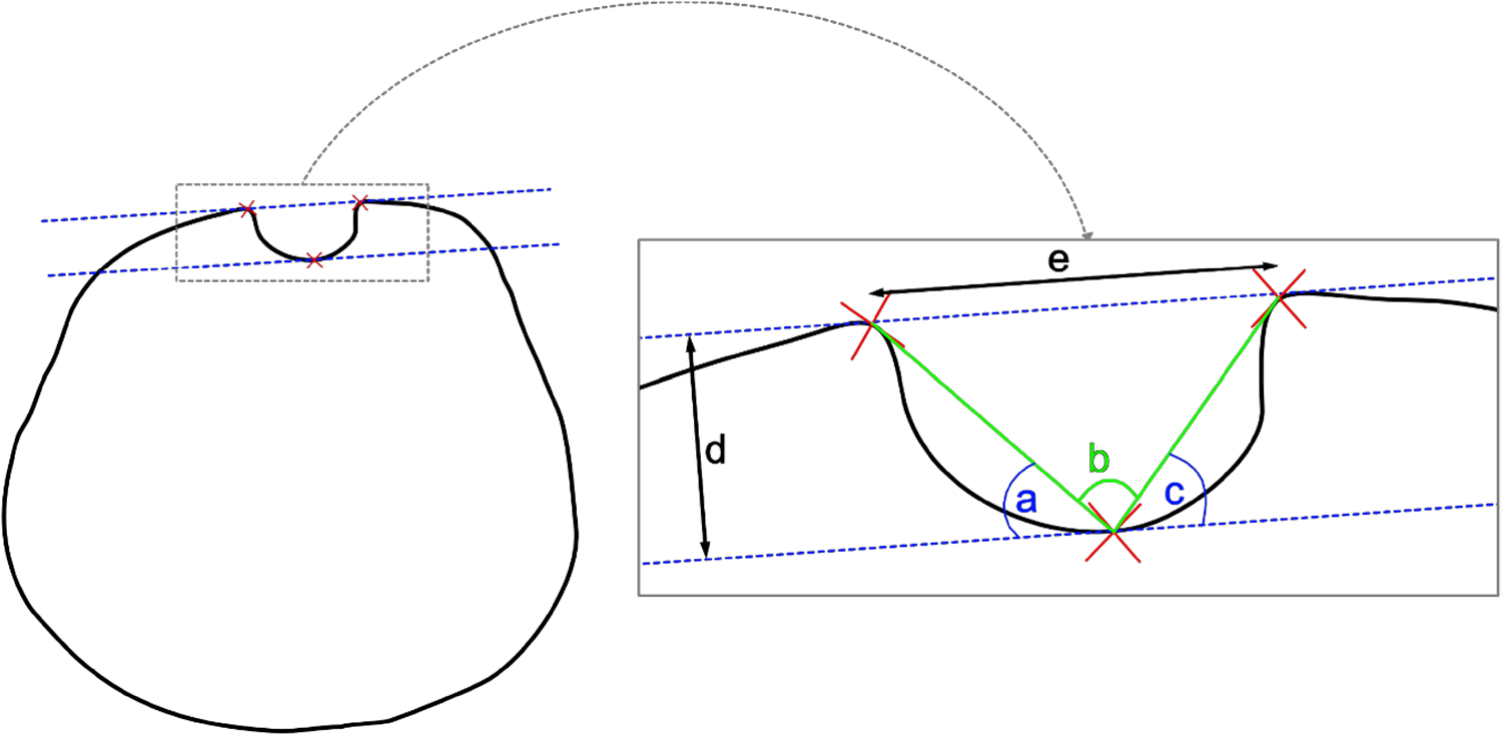
After triplanar reconstruction of the axial plane, a line was drawn between the apex of both the medial and lateral IG walls (corresponding to the lesser and greater tuberosities). A line parallel to this was then placed on the deepest point of the IG (blue dotted lines). The TOA (**b**) was then measured between the 3 landmarks (red crosses). Then the LWA (**a**) and MWA (**c**) were determined. The IG depth (**d**) was measured between the parallel lines and width (**e**) between the IG walls

MRI images were analysed using a clinical imaging software which is capable of triplanar reconstruction (OsiriX MD V12.0.3, Pixmeo SARL, Geneva Switzerland). Crossectional T2 image sets were used for analysis. Two orthopaedic registrars (BDK and JFSC) with experience in shoulder surgery independently analysed the MRIs according to a self-developed protocol. Triplanar reconstruction was carried out to measure a true cross-section of the IG at the deepest part reproducibly, whilst eliminating the problem of positioning in the MRI: The axial plane was reconstructed in 3 planes at the level of and in line with the highest points (furthest from the floor of the IG) of both the greater and lesser tuberosity and aligned perpendicular to the floor of the IG (Fig. 6). After this the depth and width of the IG, as well as the medial wall angle (MWA), lateral wall angle (LWA) and total opening angle (TOA) were measured (see Fig. 7). Measurement values were recorded accurate to one decimal place. To correct for variation in patient size, the diameter of the humeral head was measured and absolute values were expressed relative to the average humeral head diameter from this cohort.

### Statistical analysis

Statistical analysis was performed using SPSS V26.0 (IBM) statistics software. A power analysis was performed prior to study commencement. To detect a difference in TOA of 3° (from previous work), with a SD of ± 6.34 (from previous work), with a power of 80%, type 1 error of 0.05 and assuming an unequal enrolment ratio of 2–1, a necessary sample size of 159 was determined [16].

The mean of the measurement values from both observers was taken and values are displayed to 1 decimal place. Normal distribution was confirmed using the Shapiro–Wilk test. The one-way ANOVA test was employed to investigate significant differences in sample means between all subgroups. Significance was set at *p* < 0.05. Non-significant *p*-values are reported as n.s.. Where significant differences in means were confirmed using ANOVA, individual subgroup comparisons were carried out using the Tukey–Kramer test. The odds ratios (OR) for BRP injury in the presence of morphological factors was calculated, given with 95% confidence intervals (CI) and displayed to 1 decimal place. The sensitivity and specificity of a TOA > 95° for the presence of a medial or bilateral BRP lesion was calculated. Pearson’s correlation coefficient was determined to investigate a possible relationship between patient age and the TOA. The inter-class correlation coefficient (ICC) was calculated to examine the reliability of the measurements.

## Results

### Sample demographics

Of the 166 patients included in the study 51 were women, the mean age was 53 years (range 20–80). Demographics of the subgroups are shown in Table 1. The case distribution of the three types of BRP lesions are shown in Fig. 1. Forty-three patients had bilateral, 65 medial and 38 lateral BRP lesions as confirmed during arthroscopy. The 20 patients where arthroscopy found the LHBT to be stable in the absence of a BRP lesion made up the control group. Of these, 12 had tendinitis of the LHBT, 5 had a SLAP tear and 3 had a mechanical outlet impingement with a partial tear of the SSP.

**Table 1:** Demographics of the

Subgroup	Gender distribution	Age (mean)
Control group	6 of 20 female	46
Medial BRP injury	17 of 65 female	51
Lateral BRP injury	15 of 38 female	56
Bilateral BRP injury	13 of 43 female	57

*BRP* biceps reflection pulley

### IG morphology

**Fig. 8:**
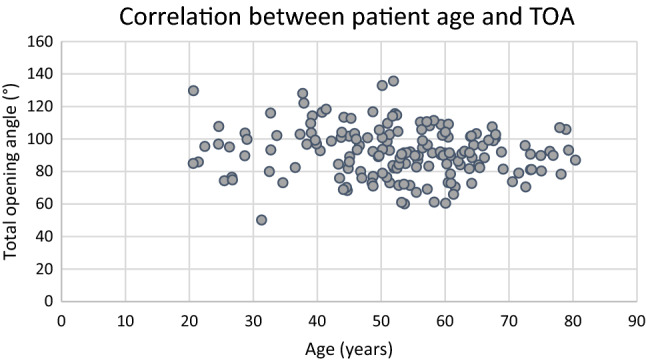
Scatter plot of the correlation between patient age and the TOA. The corresponding Pearson’s coefficient of − 0.12 showed a weak negative correlation (p = n.s.)

The ICC of the measured values showed a high level of reproducibility (Table 2). The mean humeral head diameter was 5.13 cm and absolute values were expressed relative to and this. Humeral head diameter did not differ significantly between subgroups (n.s.). The Pearson’s correlation coefficient for patient age and TOA was − 0.12 (n.s.), representing a statistically insignificant weak negative correlation, depicted in Fig. 8. When this correlation was investigated in only patients who did have a BRP injury, a statistically significant weak negative Pearson’s correlation with a coefficient of − 0.18 (*p* = 0.03) was confirmed.

**Table 2:** Inter-class correlation coefficient for the respective measurements of the intertubercular groove

Intertubercular groove measurement	Inter-class correlation coefficient
Depth	0.84
Humeral head diameter	0.96
Medial wall angle	0.92
Lateral wall angle	0.90
Total opening angle	0.93
Width	0.90

**Fig. 9:**
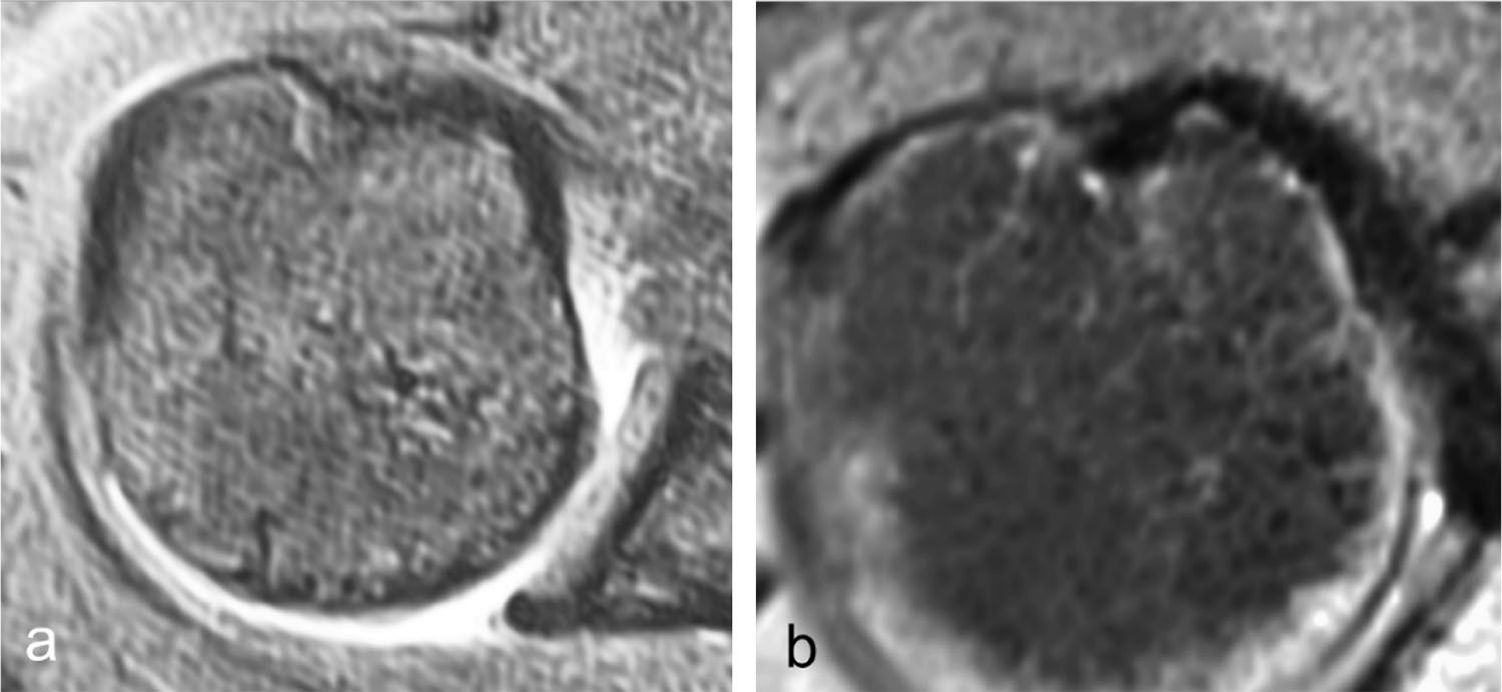
Examples of a dysplastic open and shallow IG from a patient with a bilateral BRP lesion (**a**) and an IG from a patient without a BRP lesion (**b**)

The results of the measurements of the IG morphology are displayed in Table 3. The significance of differences according to the ANOVA and Tukey–Kramer tests is shown in Table 4. Both those with medial and with bilateral BRP injuries had significantly shallower and wider IGs with a flatter MWA and wider TOA than the control group (Fig. 9). There was no difference in the morphology of the IG between patients that had a medial or bilateral BRP lesion. However, only those with bilateral BRP lesions had a significantly flatter LWA than the control group. The IG morphology of those with lateral BRP lesions did not differ significantly from the control group, whilst differing significantly from those with both medial or bilateral BRP lesions in most aspects.

**Table 3:** Mean (95% confidence interval), ± SD of the morphological IG measurements

Measurement by BRP injury type	IG Width (mm)	IG Depth (mm)	MWA (°)	LWA (°)	TOA (°)
Control group	10.8 (10.2–11.4),± 1.4	6.2 (5.9–6.5), ± 0.6	47.9 (45.5–50.3),± 5.5	49.5 (46.5–52.5), ± 6.9	82.6 (78.6–86.6), ± 9.1
Medial injury	12.5 (12.1–12.9), ± 1.4	5.5 (5.3–5.7), ± 0.97	38.8 (36.8–40.8), ± 8.4	44.9 (42.6–47.2), ± 9.4	96.1 (92.4–99.8),± 15.2
Lateral injury	11.6 (11.0–12.2), ± 1.9	6.3 (6.0–6.6),± 0.9	47.1 (44.7–49.5), ± 7.5	47.8 (45.0–50.6), ± 8.7	84.9 (80.8–89.0),± 12.8
Bilateral injury	12.3 (11.5–13.1), ± 2.6	5.4 (5.1–5.7), ± 0.9	40.0 (37.4–42.6), ± 8.7	43.3 (40.7–45.9), ± 8.8	96.6 (92.3–100.9), ± 14.4

*IG* intertubercular groove, *SD* standard deviation, *BRP* biceps reflection pulley, *MWA* medial wall angle, *LWA* lateral wall angle, *TOA* total opening angle

**Table 4:** Post hoc subgroup analysis using Tukey–Kramer significance tests between BRP injury subgroups (rows) for IG measurements (columns), after significant differences in means were confirmed for all measurements using one-way ANOVA. Significant differences (difference larger than critical *Q* value) between subgroups are highlighted in bold and marked with an asterisk

Difference in means	IG Width (mm)	IG Depth (mm)	MWA (°)	LWA (°)	TOA (°)
ANOVA p-value	0.01	< 0.001	< 0.001	0.01	< 0.001
Critical Q value	1.2	0.5	4.3	4.8	7.3
Medial injury vs control group	1.7*	0.7*	9.1*	4.6	13.6*
Lateral injury vs control group	0.7	0.1	0.4	1.1	1.3
Bilateral injury vs control group	1.5*	0.8*	7.9*	6.2*	14.0*
Medial vs bilateral injury	0.2	0.1	1.2	1.6	0.4
Lateral vs Medial injury	1.0	0.8*	8.7*	3.5	12.2*
Lateral vs bilateral injury	0.9	0.9*	7.5*	5.0*	12.7*

*MWA* medial wall angle, *LWA* lateral wall angle, *TOA* total opening angle, *IG* intertubercular groove

The OR and corresponding CI of morphological factors associated with medial or bilateral BRP lesions are shown in Table 5. The sensitivity of a TOA of > 95° for the presence of a medial or bilateral BRP lesion was 55.6% (CI 45.7%–65.1%) and the specificity was 84.5% (CI 72.6%–92.7%).

**Table 5:** Odds analysis for medial or bilateral BRP injury according to measurements of IG morphology

Morphological factor	Odds ratio (95% CI)
TOA > 95°	6.8 (3.0–15.2)
MWA < 42°	6.0 (2.8–12.8)
IG width > 13 mm	2.6 (1.2–5.6)
IG depth < 5 mm	2.4 (1.0–5.7)

*MWA* medial wall angle, *TOA* total opening angle, *IG* intertubercular groove, *CI* confidence interval

## Discussion

The most important finding of this study was that IGs of patients with medial or bilateral BRP lesions were shallower and wider, with flatter MWAs and wider TOAs than IGs of the control group, whilst IGs of patients with lateral BRP injuries did not differ from the control group. This suggests that these dysplastic IGs offer reduced stability to the LHBT, especially in the medial direction, increasing the risk of medial or bilateral BRP injury.

In the present study the OR for a medial or bilateral BRP lesion was significantly raised when a TOA of over 95° was present, which may make this a useful predictive tool in clinical practice. This factor stands out as it is a product of all the others, is easily measured and had the biggest impact in our analysis. Whilst the TOA is easily measurable in clinical practice, caution should be taken in its interpretation if measured without prior triplanar reconstruction.

Whilst the present data shows some differences in IG morphology between subgroups, the hypothesis can only be partially accepted, as lateral BRP injuries do not seem to be influenced by IG morphology. Instead of 4 types of IG morphology there seem only to be 2: The normal type, which the control group and those with lateral BRP injuries display and the dysplastic type which is common amongst those with medial or bilateral BRP lesions. This helps to explain the pathogenesis of BRP lesions, can be used as a diagnostic marker and may help to inform a decision to perform additional biceps surgery during rotator cuff repair in unclear cases.

### Interpretation

Researchers have historically described a dysplastic IG type, originally using x-ray imaging and linked this to medial dislocation of the LHBT with or without a RC tear [19]. In contrast, Abboud et al. found no significant relationship between IG morphology and tendinitis of the LHBT [1]. A more recent study investigating LHBT instability, rotator cuff tears and IG morphology also found no correlation between LHBT instability and groove morphology [28]. This is partially in contrast to our findings, although neither of these studies investigated BRP injuries as the dependent variable. Subscapularis tendon tears nevertheless did correlate with LHBT instability in the latter study, which aligns with our finding of medial and bilateral BRP injuries in the presence of a shallow IG [28]. Neither of these studies undertook a reconstruction of the investigated MRI images to allow for comparable measurements of the IG, which limits their reliability. Other MRI studies have recently shown that shallow IGs with large opening angles and flat medial angles are linked with medial dislocation of the LHBT [16, 34]. This is in line with the results of the present study, which is the first to undertake triplanar reconstruction of the IG. An analysis differentiating between BRP injury types on the basis of arthroscopy findings was carried out in this work for the first time. This revealed that lateral BRP injuries are not influenced by IG morphology, whilst medial and bilateral ones are.

The analysis of a possible correlation between patient age and IG morphology did not reveal a shallower IG with increasing age. A possible causation of a shallow IG due to secondary degeneration over time due to an instable LHBT would not be supported by this finding. For patients who had a BRP injury a statistically significant weak negative correlation between age and TOA was seen. This may be because patients with a larger TOA develop and present with BRP injuries at a younger age.

IG morphology did not differ significantly between patients with a medial BRP injury and those with a bilateral BRP injury in our study population. Given that shear force vectors acting on the LHBT have been shown to be directed medially in most physiological arm positions, it seems that the decreased stability from the IG primarily leads to medial instability of the LHBT as well as a medial injury of the BRP [7]. Considering the presence of bilateral lesions with dysplastic IG morphology, it may be that once dislocated medially the LHBT “skips” across the shallow IG during an internal rotation movement and subsequently damages the lateral BRP. Alternatively, the loss of function of the cranial SSC may increase the load placed on the ventral SSP, predisposing this to injury. Biomechanical studies are needed to investigate this further.

The sensitivity and specificity of a TOA of > 95° for predicting the presence of a BRP lesion in patients with ventral shoulder pain can be interpreted as follows: Whilst only just over half of patients who have a dysplastic IG have a medial or bilateral BRP lesion, most of those with a medial or bilateral BRP lesion have a dysplastic IG.

Concomitant LHBT surgery (tenotomy or tenodesis) during rotator cuff repair surgery is recommended routinely by some authors [2]. When degeneration of the LHBT is evident this may be necessary to alleviate pain [30]. Tenotomy alone causes reduced elbow supination strength and satisfaction when compared with tenodesis, whilst intraarticular and subpectoral tenodesis yield comparable results [3, 21]. Whilst the LHBT predominantly affects elbow function, 10% of shoulder abduction strength in external rotation can be attributed to the LHBT [29]. Additionally, it has been shown that LHBT surgery or pathology causes an upward migration of the humeral head, suggesting a depressing function of the LHBT [6, 8, 25]. Functional results after RC repair with retained anatomical insertion of the LHBT have been shown to be superior when compared to cases with additional LHBT surgery [11]. Retaining the LHBT in the shoulder joint, however, could be a cause for persistent pain or retear of the repaired RC, although this is controversial [6, 8, 9, 24]. A recent meta-analysis showed that overall concomitant LHBT surgery was a risk factor for retear after arthroscopic RC repair [35]. This could be as additional load is placed on the repaired SSP tendon if the LHBT is removed from the shoulder, although a recent biomechanical study was not able to show a significant difference regarding this [27]. This study may however have been underpowered and only tested initial shoulder abduction in one position [27]. Therefore, the decision of additional LHBT surgery during RC repair must be made on a case-by-case basis [9]. This decision could in future be informed by considering the morphology of the IG, according to the findings of the present study. Patients with a dysplastic IG may carry a higher risk for persisting instability of the LHBT and therefore a higher risk of retear of the repaired RC as well as pain. Conversely, supraspinatus tendon tears with isolated lateral pulley lesions seem not be associated with instability of the LHBT and may therefore not require concomitant LHBT surgery in patients where the LHBT is not itself degenerated [17].

As an alternative to LHBT tenotomy or tenodesis, stabilisation of the LHBT has been trialled in acute cases of BRP lesions with SSC tear [20]. Our data suggests that this should be considered only for patients without significant IG dysplasia, as those with IG dysplasia may have a higher risk of recurrence. Our data suggests a surgical deepening of the IG, analogous to the fibular groove deepening for peroneal tendon instability which is commonly performed, may potentially be of benefit for LHBT stabilisation in shoulders where the IG is dysplastic [14].

One limitation of this work is the inhomogeneous subgroup sizes. To ensure the BRP would be described in detail in the operative report, only patients with a suspected BRP lesion were retrospectively included in the study. With this study design we had no influence on the subgroup sizes. The control group (smallest) is however larger than 25% of the size of any of the other subgroups (31% at smallest), which is non-problematic statistically speaking [33].

Another limitation is that the MRIs analysed were not all performed in one centre according to a uniform protocol, as patients often presented after referral having already had MRI scans.

The operative reports, from which the types of BRP injuries were classified, were not written for scientific purposes, which could limit the accuracy with which these describe the BRP.

It can be criticised that the control group is not made up of healthy volunteers. However, as lesions of the BRP can only reliably be diagnosed or ruled out with arthroscopy, healthy volunteers are not an option for ethical reasons. Furthermore, the present comparison is clinically very relevant, as the subgroups are made up of patients which may present with a similar clinical picture.

The results of this study can help inform a decision to perform concomitant LHBT surgery during rotator cuff repair, which may not be necessary when an isolated lateral BRP injury with SSP tendon tear is present.

## Conclusions

A dysplastic type of IG morphology with a wide TOA, flat MWA, decreased depth and increased width is associated with the presence of medial and bilateral BRP injuries. A TOA of > 95° increases the likelihood of a medial or bilateral BRP injury 6.8-fold. Lateral BRP injuries are not associated with dysplastic IG morphology. Concomitant LHBT surgery may therefore not always be necessary during isolated SSP tendon repair.

## Data Availability

The datasets generated during and/or analysed during the current study are not publicly available due to institutional data protection agreements but are available from the corresponding author on reasonable request.
